# Incidence and risk factors for vaso occlusive crisis among children living with sickle cell disease in Uganda: a cohort study

**DOI:** 10.21203/rs.3.rs-9665061/v1

**Published:** 2026-05-26

**Authors:** Robert Kitenge, Joseph Rujumba, Bernard kikaire, Deogratias Munube, Sarah Kiguli, Ruth Namazzi

**Affiliations:** Makerere University College of Health Sciences; Makerere University College of Health Sciences; Makerere University College of Health Sciences; Mulago National Referral Hospital; Makerere University College of Health Sciences; Makerere University College of Health Sciences

**Keywords:** Sickle Cell Anaemia, Vaso-Occlusive Crisis, Incidence, Risk Factors, Children, Uganda

## Abstract

**Background:**

Vaso-occlusive crises (VOC) are the most frequent complications among people living with sickle cell disease. VOC lead to frequent hospitalisations, reduce quality of life and contribute to long-term sequelae such as stroke, acute chest syndrome, and renal impairment. There are limited contemporary data on the incidence and risk factors for VOC among children with sickle cell anaemia (SCA) after the introduction of hydroxyurea in Uganda.

**Methods:**

A prospective cohort study conducted among children living with SCA enrolled in the Uganda Sickle Pan-African Research Consortium Registry, at the Mulago Hospital Sickle Cell Clinic. Participants aged 6 months to 18 years enrolled and followed for six months to assess the occurrence of VOC. Cox proportional hazards regression was used to evaluate associations between clinical and laboratory variables and time to VOC.

**Results:**

A total of 438 participants were enrolled, with a median age of 9 years (IQR: 5–13). Most participants (80.1%) reported hydroxyurea use. The mean baseline haemoglobin concentration was 7.7 g/dL (SD: 1.2), and the median foetal haemoglobin (HbF) level was 12.3% (IQR: 7.2–18.2). in the preceding year, history of VOC was reported by 88.8%, and 30.1% had received at least one blood transfusion. The incidence of VOC was 102.3 (95% CI: 88.2–118.8) per 100 person-years, with 13% experiencing more than one episode during follow-up. After adjusting for sex and prior VOC history, previous blood transfusion was independently associated with increased VOC risk (aHR: 1.11; 95% CI: 1.02–1.22; *p* = 0.020).

**Conclusion:**

VOC remains a common and clinically significant complication among Ugandan children living with SCA. Prior blood transfusion was identified as a potential predictor of VOC, underscoring the need for optimised clinical management, including timely and appropriately dosed hydroxyurea therapy, to mitigate risk in this population.

## Introduction

Sickle cell disease (SCD) is the most prevalent monogenic disorder globally(1), with an estimated 300,000 infants born annually with sickle cell anaemia (SCA), a number projected to increase to approximately 400,000 by 2050(2). Recognising its significant public health burden, the World Health Organisation (WHO) designated SCA a global health priority in 2006(3). Sub-Saharan Africa bears the brunt of this burden, accounting for approximately 75% of global SCA births, with an estimated 230,000 affected infants in 2010 alone (4). The disease contributes substantially to early childhood mortality in the region, with reported mortality rates ranging from 50% to 90% before the age of five (5–7). In Uganda, data from neonatal screening studies indicate a prevalence of 0.7% for sickle cell disease and 13.3% for the sickle cell trait among newborns (8).

Sickle cell anaemia results from the homozygous inheritance of the HbS allele, leading to structural abnormalities in red blood cells that predispose them to sickling under hypoxic conditions. (9) The clinical course of SCA is marked by both acute complications, such as vaso-occlusive crisis (VOC), acute anaemia, and recurrent infections, and chronic sequelae, including haemolytic anaemia, end-organ damage, glomerulopathy, pulmonary hypertension, and cerebral vasculopathy, (6). VOC, the hallmark of SCA, arises from a complex pathophysiological process involving not only microvascular obstruction by sickled erythrocytes but also large-vessel intimal hyperplasia, thrombosis, and bone marrow fat embolization(10). Pain from VOC commonly affects the lower back, joints, and extremities (11) and represents the leading cause of hospital admissions among SCA patients (12). Multiple factors have been implicated in VOC frequency, including foetal haemoglobin levels, lactate dehydrogenase concentrations, leukocyte count, blood viscosity, infections, wind speed, cold, rain and air pollution. (13–15).

In recent years, notable advancements have been made in the management of SCA in Uganda, including the introduction and increasing uptake of hydroxyurea therapy, which has been shown to reduce the frequency and severity of VOC (16,17). Despite these improvements, VOC continue to be the predominant reason for hospitalisation among children with SCA (18). Data on the incidence and associated risk factors for VOC in the current treatment era, however, remain sparse, particularly in Uganda, where healthcare infrastructure and access to disease-modifying therapies remain inconsistent. A recent study conducted in Eastern Uganda reported an incidence of 190 VOC events per 100 child-years among young children with SCA (19), underscoring the persistent burden of this complication.

VOC is not only contribute to frequent hospitalisations but also negatively impact the quality of life of affected children, leading to school absenteeism, psychosocial distress, and long-term complications (20–22). Moreover, while hydroxyurea is available as a standard-of-care intervention, its accessibility remains suboptimal; stockouts in public clinics often force families to purchase the medication from private pharmacies, posing financial challenges.

The burden of VOC in Uganda, particularly in the paediatric population, is not well documented. Understanding the incidence and risk factors associated with VOC in the context of current clinical practices, including hydroxyurea use, is essential to informing policy and clinical decision-making. This study was therefore conducted to estimate the incidence of and identify risk factors for vaso-occlusive pain crisis among children with SCA attending the Mulago Sickle Cell Clinic to guide strategies to mitigate disease burden and improve health outcomes in this high-risk population.

## Methods

### Study design and participants

We conducted a prospective cohort study to estimate the incidence of vaso-occlusive crises (VOC) and identify risk factors among children living with sickle cell disease (SCD) receiving care at Mulago National Referral Hospital (MNRH), Kampala, Uganda. Participants were enrolled between August 2024 and March 2025 and followed for at least 6 months. Participants were selected from the SPARCO registry using stratified sampling based on three predefined age categories: <5, >5–12, and >12–18 years. The sampling process began with the stratification of participants into these age groups. A proportionate number of participants were then selected from each age stratum by calculating the required sample size for each group, which was determined by multiplying the total sample size by the proportion of participants in each stratum. Simple random sampling was employed within each stratum to ensure unbiased selection, with random numbers generated using Stata software version 15. Once selected, eligible participants, along with their parent(s) or guardian(s), were invited to the clinic for the informed consent/assent process and enrolment, in accordance with ethical guidelines.

Eligible participants were aged 6 months to 18 years, and had a confirmed diagnosis of SCD with electrophoresis of haemoglobin, and were registered in the Uganda site of the Strengthening Capacity for Clinical Care, Research, and Training for Sickle Cell Disease (SPARCO) initiative.

Exclusion criteria were acute pain at enrolment, or any condition likely to interfere with participation for example missing phone number. Written informed consent was obtained from parents or legal guardians; and assent was obtained from children aged ≥7 years.

### Setting

The study was conducted at the Mulago Sickle Cell Clinic (MSCC), located within Mulago National Referral Hospital in Kampala, Uganda. The clinic operates five days per week and serves approximately 250 patients weekly, of whom about 80% are children aged 6 months to 18 years. Care is provided by a multidisciplinary team including paediatric haematologists, fellows, residents, medical officers and clinical officers. VOC are managed on an outpatient basis; patients requiring further care are admitted to the inpatient ward.

### Sample size and sampling

Sample size was estimated using Peruzzi’s formula for Cox proportional hazards regression, assuming 15 predictor variables and outcome proportion of 0.65 from Baby Hug trial (23), giving a minimum of 429 participants. Allowing for 10% attrition, the target sample size was 472.

Participants were selected from the SPARCO registry using stratified random sampling by age group (<5 years, 5–12 years, 12–18 years). Random numbers were generated in Stata version 15.0 to ensure unbiased selection.

### Variables

The primary outcome was the occurrence of ≥1 VOC during follow-up. Independent variables included sociodemographic characteristics (age, sex, caregiver education and employment), clinical history (number of VOC episodes in the previous year, length of recent admissions, vaccination status, prophylactic therapy use) and baseline laboratory measures (white cell count, haemoglobin, fetal haemoglobin, reticulocyte count, lactate dehydrogenase).

### Data collection and management

Secondary data were extracted from the SPARCO registry using a pretested abstraction form. Primary data on VOC episodes were collected via structured caregiver interviews during clinic visits and hospital admissions, and from patient-held medical records. Data were entered daily into Excel, double-checked, and cleaned weekly in Stata.

Quality control measures included double data entry, cross-checking with source documents, training of research assistants, and piloting of study tools. Instruments were translated into Luganda to ensure comprehension.

### Statistical analysis

Continuous variables were summarised as mean (SD) if normally distributed, or median (IQR) if skewed. Categorical variables were presented as counts and percentages. Time to first VOC was analysed using Kaplan–Meier estimates, with survival curves compared using the log-rank test. Cox proportional hazards regression identified factors associated with VOC. Variables with p<0.20 in univariable analysis, those with biological plausibility, or reported in prior literature were entered into multivariable models. Predictors with p<0.05 and known confounders were retained.

### Ethics

Ethical approval was obtained from the School of Medicine Research and Ethics Committee (Mak-SOMREC-2024–939), Makerere University, and administrative clearance from MNRH and SPARCO. Written informed consent was obtained from parents or guardians; assent was obtained from children where applicable. Confidentiality was maintained through anonymisation and secure storage of study documents.

## Results

A total of 461 children were included in the study from the SPARCO database after 23 out of the 438 screened children were excluded due to having a vaso-occlusive crisis (VOC) at enrolment, with missing data. Of these, 265 had no VOC, and 173 had VOC with in the 6 months.

### Participants’ sociodemographic, clinical and laboratory characteristics

A total of 438 children with confirmed HbSS genotypes were enrolled at Mulago National Referral Hospital between August 2024 and September 2025. The participants were stratified by age into three groups: 0.5–5.0 years (30.8%), 5.1–10.0 years (30.8%), and 10.1–18.0 years (38.4%), with a median age of 9 years (IQR: 5–13).

Clinical assessment revealed that 69.0% (302/438) of the eligible children were unvaccinated for Pneumococcus booster. A history of vaso-occlusive crisis (VOC) within the past year was reported by 88.8% (389/438), with a median of 2 episodes (IQR: 1–3).

The laboratory findings at enrolment revealed leucocytosis (WBC >12.0 ×10^9^/L) in 58.9% (258/438) and thrombocytosis (platelets >407 ×10^9^/L) in 44.5% (195/438) of the patients. The mean haemoglobin concentration was 7.7 g/dL (SD: 1.2), with 91.1% (399/438) having values within the range of 6.1–12.4 g/dL. Further details of the characteristics are presented in Table

Incidence rate of VOC among the study participants

The cumulative follow-up duration amounted to 169.07 person-years, with 173 new cases of VOC documented. The incidence rate of VOC among study participant was 102.32 (95% CI: 88.16–118.77) per 100 person-years. Only 39.5% of the participants developed VOC over the study period.

### Factors associated with VOC

#### Unadjusted analysis

According to the unadjusted analysis, a higher transfusion frequency was significantly associated with an increased risk of vaso-occlusive crisis (VOC) (HR: 1.12; 95% CI: 1.02–1.22; *p* = 0.012). Caretaker education at the ≥A level (HR: 1.42; 95% CI: 0.95–2.10; *p* = 0.084) and the presence of comorbidities (HR: 1.50; 95% CI: 0.99–2.27; *p* = 0.058) showed positive trends but did not reach statistical significance. Other factors, including sex, hydroxyurea use, vaccination status, and laboratory parameters, were not significantly associated with VOC occurrence.

### Adjusted analysis

In the adjusted analysis, transfusion frequency remained significantly associated with an increased risk of VOC (aHR: 1.11; 95% CI: 1.02–1.22; p = 0.020). No other covariates showed statistically significant associations. Further details are provided in Table 3.

### Survival curve categorised by transfusion status at enrolment

**Figure 1:**
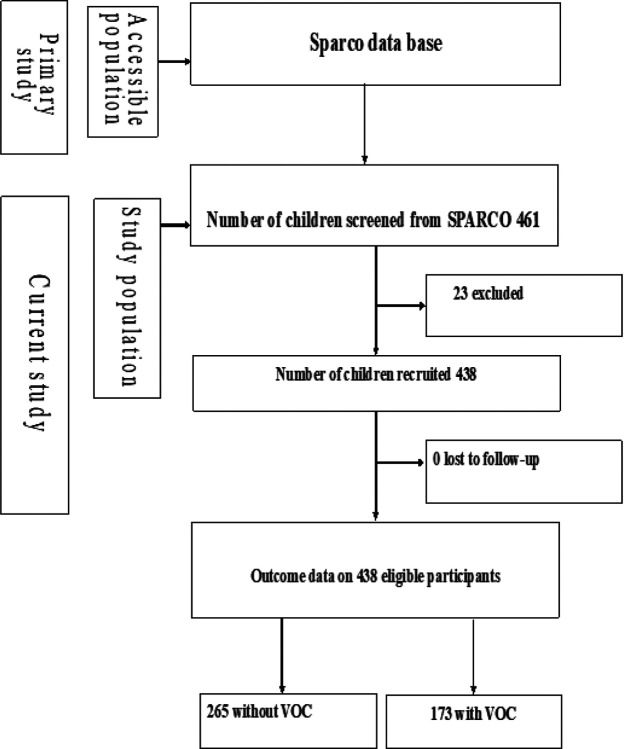
Participant flow diagram displaying the recruited participants and the number of participants analysed

Figure 1 illustrates event-free survival probabilities for VOC among children aged 6 months to 18 years at Mulago National Referral Hospital, stratified by transfusion status between August 2024 and March 2025. Compared with their nontrans fused counterparts, children who received transfusions had significantly lower VOC-free survival.

## Discussion

This prospective cohort study assessed the incidence and risk factors for vaso-occlusive crisis (VOC) among children living with sickle cell disease (SCD) at Mulago National Referral Hospital. Over a follow-up period of 169.07 person-years, 173 VOC events were recorded, yielding an incidence rate of 102.32 per 100 person-years (95% CI: 88.16–118.77). Approximately 40% of the participants experienced at least one VOC, indicating a substantial disease burden. Blood transfusion frequency emerged as a significant predictor of VOC (HR: 1.12; *p* = 0.012).

The incidence rate observed is higher than that reported in the multicentre REACH trial (44.6 per 100 person-years)(16), potentially because of the predominance of the severe HbSS genotype, possibly compounded by the Bantu haplotype(5), limited hydroxyurea access, poor adherence, and suboptimal dosing, although these factors were not directly assessed. Conversely, the VOC incidence in this study was lower than that reported in the Zinc trial conducted in Jinja (190 per 100 person-years), likely due to the Zinc trial's enrolment of patients with more severe disease profiles, such as frequent prior VOC and baseline haemoglobin < 6 g/dL (19).

Blood transfusion frequency emerged as a significant predictor of VOC. This association likely reflects more severe underlying disease or inadequate disease-modifying therapy, reinforcing the role of transfusion history as a clinical marker for VOC risk stratification. Although not statistically significant, the presence of comorbidities and higher caregiver education showed suggestive associations. The counterintuitive trend with caregiver education may indicate increased health-seeking behaviour or improved symptom recognition and reporting among more educated caregivers. Previous studies have identified lower caregiver education as a risk factor for worse SCD outcomes(24, 25), underscoring the need for further qualitative research into caregiver perceptions and reporting behaviours.

Children with comorbidities such as stroke, acute chest syndrome, and priapism are at increased risk for VOC, which aligns with the known pathophysiology of systemic inflammation and vascular dysfunction in severe SCD. Stroke, accounting for 67.3% of the comorbidities in this cohort, further highlights the need for targeted neuroprotective strategies. While laboratory markers such as haemoglobin, LDH, and white cell count did not correlate with VOC incidence in this study, as reported in higher-income settings(13, 26), this may reflect differences in clinical monitoring practices or resource limitations.

Although the effectiveness of hydroxyurea has been demonstrated in both high-resource trials (e.g., BABY-HUG) (23) and African settings (e.g., REACH trial), (16) its limited observed impact in this cohort suggests implementation challenges, including inconsistent access, subtherapeutic dosing, and adherence.

The strengths of this study include its prospective design, large sample size, and inclusion of clinical and laboratory variables, which collectively increase the validity and depth of the findings. However, the relatively short follow-up duration may have limited the detection of long-term or seasonal VOC patterns. Additionally, reliance on self-reported medication use without adherence verification may have underestimated the impact of hydroxyurea, although biomarker data (HbF and LDH) provided some objective evidence of treatment exposure.

## Conclusion and recommendations

This study revealed a substantial burden of VOC among children with sickle cell disease (SCD) at Mulago National Referral Hospital. Increased blood transfusion frequency was significantly associated with a greater risk of VOC, suggesting its potential as a marker of disease severity and a predictor of VOC.

To address this burden, preventive strategies should be strengthened, including improved access to hydroxyurea therapy, a consistent drug supply, and enhanced caregiver education to support adherence. A history of transfusion may help identify high-risk patients who could benefit from early initiation and dose escalation of hydroxyurea. Given the limited follow-up period in this study, future research should involve longer-term cohort studies to identify additional risk factors and inform more targeted and effective interventions for VOC prevention and SCD management.

## Figures and Tables

**Figure 2:**
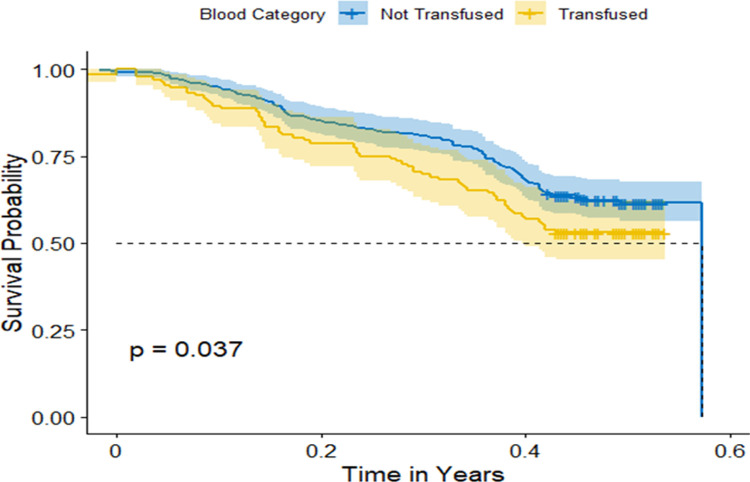
Figure 1. Presents probabilities of not developing VOC among participants, stratified by transfusion status.

**Table 1:** Sociodemographic, clinical, and laboratory characteristics of participants at Mulago.

Variable	Category	Frequency (%)
		N=438
**Sociodemographic**		
**Age**	Median (IQR)	9 (5, 13)
	1–5	135 (30.8)
	5.1–10	135 (30.8)
	10.1–18	168 (38.4)
**Sex**	Male	225 (51.4)
	Female	213 (48.6)
**Highest Education Level**	≤ Primary	143 (32.7)
	O-level	190 (43.4)
	≥ A-level	105 (24.0)
**Employment Status**	Unemployed	33 (7.5)
	Employed	405 (92.5)
**Clinical characteristics**		
Blood transfusion*	Yes	132 (30.1)
Vaccination Status*	Yes	136 (31.1)
VOC history*	Yes	389 (88.8)
**Hydroxyurea use**	Yes	354 (80.8)
Comorbidities*	Yes	52 (11.9)
**Penicillin V use**	Yes	428 (97.7)
**Fansidar use**	Yes	436 (99.5)
**Splenectomy history**	Yes	2 (0.5)
**Laboratory characteristics**		
**Leucocyte count**	Median (IQR)	12.6 (10.2, 15.2)
	≤10.0	180 (41.1)
	>10.0	258 (58.9)
**Platelet count**	Median (IQR)	407 (324, 506)
**Haemoglobin (g/dl)**	Mean (Sd)	7.7 (1.2)
	3.1–6.0	39 (8.9)
	6.1–12.4	399 (91.1)
**Reticulocyte count**	Mean (Sd)	309.6 (234.3,393.2)
	5–556	249 (56.9)
	557–2471	189 (43.2)
**Haemoglobin F**	Median (IQR)	12.3 (8.2, 16.7)
	0.6–10	140 (32.0)
	10.1–39.8i	298 (68.0)
**LDH**	Median (IQR)	556 (463, 658)
	5–500	140 (32.0)
	501–2471	298 (68.0)

* vaccination status: reflects receipt of the pneumococcal booster dose

* history of voc in the past year: history of voc in 2024 prior to enrolment

* existence of comorbities: comorbidities include history of stroke, priapism, or acute chest syndrome before enrolment

* history of blood transfusion: history of blood transfusion refers to the number of transfusion episodes during 2024 prior to enrolment

**Table 2.** Unadjusted and adjusted relationships between explanatory factors and VOC among participants.

Variable	Category	Unadjusted HR	
		HR (95% CI)	P value
**Sociodemographic**			
**Sex**	Male	1	
	Female	1.23 (0.91, 1.67)	0.171
**Highest Education Level**	≤ Primary	1	
	O-level	1.19 (0.83, 1.69)	0.348
	≥ A-level	1.42 (0.95, 0.21)	0.084
**Employment Status**	Unemployed	1	
	Employed	1.00 (0.57, 1.76)	0.998
**Clinical characteristics**			
**Transfusion**	Frequency	1.12 (1.02, 1.22)	0.012
**Vaccination Status**	No	1	
	Yes	0.87 (0.62, 1.21)	0.397
**VOC history**	No	1	
	Yes	1.25 (0.75, 2.10)	0.388
**Hydroxyurea Use**	No	1	
	Yes	1.08 (0.74, 1.59)	0.695
**Comorbidities**	No	1	
	Yes	1.50 (0.99, 2.27)	0.058
**Laboratory characteristics**			
**Leucocyte Count**	3.0–12.0	1	
	12.01–43.2	1.04 (0.76, 1.41)	0.818
**Platelet Count**	9–407	1	
	408–1495	1.02 (0.76, 1.38)	0.878
**Haemoglobin (g/dl)**	3.1–6.0	1	
	6.1–12.4	1.04 (0.61, 1.77)	0.876
**Reticulocyte Count**	5–556	1	
	557–2471	0.97 (0.61, 1.53)	0.886
**Haemoglobin F**	0.6–10	1	
	10.1–39.8	1.21 (0.87, 1.69)	0.251
**LDH**	5–500	1	
	501–2471	1.13 (0.82, 1.56)	0.462

**Table 3.** Adjusted relationships between explanatory factors and VOC among participants.

Variable	Category	Adjusted HR	
		AHR (95% CI)	P value
**Transfusion**	Frequency	1.11 (1.02, 1.22)	0.020
**Sex**	Male	1	0.284
	Female	1.18 (0.87, 1.59)	
**VOC history**	No	1	0.433
	Yes	1.23 (0.73, 2.06)	

## Data Availability

All the data generated/analysed during this study are included in this manuscript. The datasets generated and/or analysed during the study are also available from the author upon request.
